# Mechanism of Glucose Water as a Neural Injection: A Perspective on Neuroinflammation

**DOI:** 10.3390/life12060832

**Published:** 2022-06-02

**Authors:** Yung-Tsan Wu, Yen-Po Chen, King Hei Stanley Lam, Kenneth Dean Reeves, Jui-An Lin, Cheng-Yi Kuo

**Affiliations:** 1 Department of Physical Medicine and Rehabilitation, Tri-Service General Hospital, School of Medicine, National Defense Medical Center, Taipei 11490, Taiwan; crwu98@gmail.com; 2 Integrated Pain Management Center, Tri-Service General Hospital, School of Medicine, National Defense Medical Center, Taipei 11490, Taiwan; 3 Department of Research and Development, School of Medicine, National Defense Medical Center, Taipei 11490, Taiwan; 4 Department of Animal Science, The iEGG and Animal Biotechnology Research Center, National Chung Hsing University, Taichung 402, Taiwan; chenyp@dragon.nchu.edu.tw; 5 The Hong Kong Institute of Musculoskeletal Medicine, Hong Kong; drlamkh@gmail.com; 6 Department of Family Medicine, The Chinese University of Hong Kong, Hong Kong; 7 Department of Family Medicine, The University of Hong Kong, Hong Kong; 8 Center for Regional Anesthesia and Pain Medicine, Wan Fang Hospital, Taipei Medical University, Taipei 116, Taiwan; juian.lin@tmu.edu.tw; 9 Private Practice PM&R and Pain Management, Roeland Park, KS 66205, USA; deanreevesmd@gmail.com; 10 Department of Anesthesiology, School of Medicine, College of Medicine, Taipei Medical University, Taipei 110, Taiwan; 11 Department of Anesthesiology, Wan Fang Hospital, Taipei Medical University, Taipei 116, Taiwan; 12 Pain Research Center, Wan Fang Hospital, Taipei Medical University, Taipei 116, Taiwan; 13 Department of Anesthesiology, School of Medicine, National Defense Medical Center, Taipei 11490, Taiwan; 14 Department and Graduate Institute of Biology and Anatomy, National Defense Medical Center, Taipei 11490, Taiwan

**Keywords:** glucose, neuroinflammation, cytokine, nerve

## Abstract

The entrapment of peripheral nerves is associated with chronic neuroinflammation and neuropathic pain, and perineural injection therapy with glucose is emerging as an effective treatment for peripheral entrapment neuropathy. However, the mechanism underlying the pharmacological effect of glucose on nerves remains unclear. One of the hypothesized mechanisms is that glucose reduces neurogenic inflammation. Therefore, we investigated the effects of high glucose concentrations on cytokine-induced neuroinflammation in vitro. Human SH-SY5Y neuronal cells were challenged with 10 ng/mL TNF-α for 16 h and subsequently treated with different glucose concentrations (0–25 mM) for 24 h. Cell viability was evaluated using the diphenyltetrazolium bromide assay, and proinflammatory cytokine levels were assessed using ELISA and quantitative PCR. In addition, mRNA levels of NF-κB and cyclooxygenase-2 were analyzed using quantitative PCR. Exposure to 10 ng/mL TNF-α resulted in decreased viability of SH-SY5Y cells and significant upregulation of IL-6, IL-1β, NF-κB, and cyclooxygenase-2. Subsequent exposure to high glucose levels (25 mM) markedly reduced the upregulation of IL-6, IL-1β, cyclooxygenase-2, and NF-κB, and restored the functional metabolism of SH-SY5Y cells, compared with that of the normal glucose control. Our findings suggest that high glucose concentrations can mitigate TNF-α-induced NF-κB activation, upregulation of proinflammatory cytokines, and metabolic dysfunction.

## 1. Introduction

The entrapment of peripheral nerves results in the interruption of nerve microcirculation, ischemia, impaired nerve conduction, and alteration of nerve dynamics due to nerve adhesion and inflammation, which sequentially causes numbness, paresthesia, neuropathic pain, or muscle atrophy and weakness [1,2]. On a worldwide basis, disabling upper and lower extremity entrapment syndromes are particularly common, with carpal tunnel syndrome (CTS) and cubital tunnel syndromes having annual incidence rates of 276/100,000 [3] and 25/100,000 [4] cases, respectively. Palpation-guided subcutaneous perineural injection therapy, which involves the injection of glucose water to reduce neuropathic pain, was first reported by John Lyftogt in 2007 [5]. Over the next decade, this technique was modified by Lam et al. [6,7,8,9] and Wu et al. [10] to incorporate ultrasound guidance to hydrodissect peripheral nerves to treat entrapment neuropathy and neuropathic pain [11]. Technique improvements have been followed by a rapid expansion of the supportive literature concerning hydrodissection with 5% glucose water, including multiple level I–II randomized controlled trials [10,12,13,14,15,16]. This neural intervention is now listed as one of the treatment options by UpToDate [17] and the 20th edition of Harrison’s Principles of Internal Medicine for CTS [18]. A recent review also provides insight into the use of hydrodissection with 5% glucose water, including preliminary evidence for its benefit in the treatment of other peripheral entrapment neuropathies [19]. As chronic pain and compression neuropathy treatment with therapeutic glucose injections is an increasingly common area of high-level clinical research, it is important to search for a basic science mechanism. Recent basic science studies on the physiological effects of glucose injections have revealed potential benefits from glucose intravesical injections in an in vivo murine model of interstitial cystitis in the form of altered behavior and bladder wall regeneration [20], in vitro evidence for glucose stimulation of cartilage deposition by chondrogenic cells [21], and upregulation of favorable cytokines in an in vivo study on changes in human knee osteoarthritis, which demonstrated a significant association between hypertonic glucose treatment for osteoarthritis and upregulation of MMP2, TIMP-1, EGF, CXCL9, and IL-22 [22].

The mechanism of hydrodissection with 5% glucose water for peripheral entrapment neuropathy can be divided into mechanical and pharmacological effects. Detaching the nerve by a non-specific effect of fluid under force may progressively lessen adhesions, increase blood flow, and remobilize the nerve for neuroregeneration [6,7,11,23,24]. However, understanding the superior effects of glucose as an injectate for hydrodissection requires further understanding of its pharmacological mechanism, which is the focus of our study. Several researchers have proposed that glucose elevation, via a potential sensorineural mechanism, stabilizes neural activity or decreases neurogenic inflammation, thereby reducing neuropathic pain. When the nerve is in a hypoglycemic environment, histopathological changes result in peripheral nerves, along with the activation of nociceptive C-fibers, with increased noxious signal transduction [25,26]. If glucose is added, the excessive activation of nociceptive C-fiber nerves quickly returns to normal [25]. Moreover, elevated extracellular glucose concentrations can hyperpolarize C-fibers to stabilize their activation [27]. Although some cell lines, such as human chondrocytes or monocytes, react adversely to high glucose levels (25 mM) by expressing proinflammatory cytokines and become increasingly dysfunctional [28,29], neurons and neuronal stem cells appear to tolerate and even require higher glucose levels, up to 40 mM [30,31,32,33,34], and react much more adversely to low glucose than low oxygen concentrations [35]. In this study, we explored the effects of glucose on human SH-SY5Y neuroblastoma cells in the presence of a proinflammatory mediator, tumor necrosis factor-alpha (TNF-α), which correlates with neuropathic pain in constrictive neuropathy [36]. We hypothesized that glucose would exert an anti-inflammatory effect by reducing TNF-α-induced inflammatory mediator levels in vitro.

## 2. Materials and Methods

### 2.1. Cell Culture

Human SH-SY5Y neuroblastoma cells (CRL-2266, American Type Culture Collection ATCC, Manassas, VA, USA), a commonly studied neuronal cell type, are widely used for in vitro studies of many neurological disorders, including neuroinflammatory and neurotoxicity models, because SH-SY5Y cells have similar characteristics to nerve cells [37,38,39]. SH-SY5Y cells were grown in Dulbecco’s modified Eagle medium (DMEM)/F12 Hams with 17.5 mM glucose and supplemented with 2 mM L-glutamine, 100 U/mL penicillin, 100 μg/mL streptomycin (Life Technologies Inc., Carlsbad, CA, USA), and 10% (*v*/*v*) fetal bovine serum (Invitrogen, Waltham, MA, USA). Cells were incubated at 37 °C in a humidified atmosphere containing 5% CO_2_ and 95% air. The culture medium was changed every 2–3 days.

**Figure 1:**
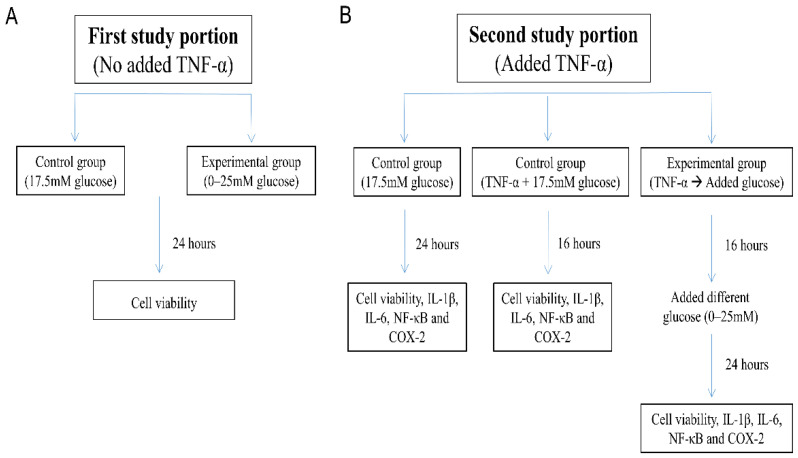
Flow chart of the study. (**A**) First study portion evaluated the effect of glucose on the absence of TNF-α in the viability of SH-SY5Y cells. (**B**) Second study portion evaluated the effect of glucose in the presence of TNF-α on cellular viability and the levels of IL-1β, IL-6, NF-κB, and COX-2.

The first part of the study evaluated the effect of glucose on the absence of TNF-α in the viability of SH-SY5Y cells. Cells that had been maintained in DMEM/F12 Hams with 17.5 mM glucose were washed and harvested for exposure to DMEM/F12 Hams medium with varying glucose concentrations (0–25 mM) (Figure 1A).

The second part of the study evaluated the effect of glucose in the presence of TNF-α on cellular metabolic activity and the levels of IL-1β, IL-6, nuclear factor kappa B (NF-κB), and cyclooxygenase type 2 (COX-2). SH-SY5Y cells were first challenged with TNF-α (10 ng/mL) to mimic the inflammatory environment, as in a previous study [40]. Three data points were available for each evaluated item (Figure 1B):

1. “17.5 mM glucose control”: SH-SY5Y cells were maintained for 24 h in DMEM/F12 Hams with 17.5 mM glucose;
2. “TNF-α + 17.5 mM glucose control”: SH-SY5Y cells exposed to 10 ng/mL TNF-α for 16 h in DMEM/F12 Hams with 17.5 mM glucose; and
3. “TNF-α → Added glucose groups”: Washed and harvested SH-SY5Y cells were first pretreated with TNF-α (10 ng/mL) in 1% FBS DMEM/F12 Hams without glucose for 16 h [34], followed by incubation for an additional 24 h with glucose added to the culture medium at 0–25 mM concentration.

### 2.2. Cell Viability Assay

A diphenyltetrazolium bromide (MTT) assay was performed to determine the cell viability, as measured by the metabolic activity of cells. A total of 1 × 10^4^ cells were seeded in each well of a 96-well plate and cultured overnight before treatment. After treatment, the culture medium was aspirated, and 0.5 mg/mL MTT solution (Sigma-Aldrich, Saint Louis, MO, USA) was added to each well before incubation for 3 h at 37 °C. The supernatant was aspirated, and dimethyl sulfoxide (Sigma-Aldrich) was added to release and form acriflavine. The resulting absorbance was measured at 540 nm using the EMax^®^ (Endpoint ELISA Microplate Reader; Molecular Devices Inc., San Jose, CA, USA).

### 2.3. ELISA

Cell culture media were collected and centrifuged at 13,500× *g* for 10 min at 4 °C, and the resulting supernatants were stored at −80 °C for subsequent analyses. The levels of IL-1β and IL-6 in the culture medium were measured using an enzyme-linked immunosorbent assay (ELISA) kit (R&D Systems Inc., Minneapolis, MN, USA).

### 2.4. Quantitative Real-Time PCR

After treatment, total RNA was extracted using the TRIzol reagent (Ambion, CA, USA). The amount of RNA sample was determined using the Qubit^®^ RNA Assay Kit (Invitrogen, Carlsbad, CA, USA) with the primer pairs listed in Table 1. Reverse transcription was performed in a 20 μL reaction with 200 ng total RNA using a high-capacity cDNA reverse transcription kit (Applied Biosystems, Carlsbad, CA, USA). Relative quantification of apoptosis and autophagy markers was performed using an ABI 7900HT system (Applied Biosystems). The housekeeping gene β-actin was used as an internal control. The measured genes included IL-1β, IL-6, NF-κB, and COX-2.

### 2.5. Statistical Analysis

All collected data were statistically analyzed using IBM SPSS Statistics version 22. Data are presented as mean ± SD of three independent experiments. One-way ANOVA by Bonferroni post hoc tests was performed to evaluate the between-group differences for significance. Repeated measures ANOVA was also performed between different doses of glucose within the same group. Statistical significance was set at *p* < 0.05.

## 3. Results

### 3.1. Effects of Glucose on the Metabolic Activity of SH-SY5Y Cells

**Figure 2:**
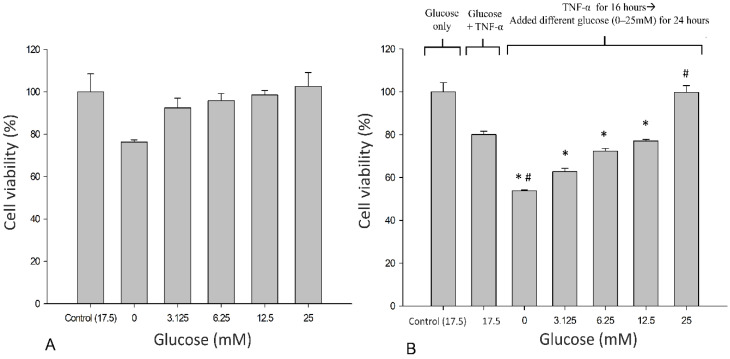
Effects of glucose on the viability of neuronal SH-SY5Y cells. (**A**) SH-SY5Y cells were treated with glucose at different concentrations for 24 h. (**B**) SH-SY5Y cells were exposed to TNF-α (10 ng/mL) and treated with different concentrations of glucose for 24 h. Cell viability was determined using the MTT assay. Data are expressed as mean ± SD. * *p* < 0.05 compared with 17.5 mM glucose control; ^#^ *p* < 0.05 compared with TNF-α + 17.5 mM glucose control.

Neuronal SH-SY5Y cells were treated with glucose concentrations of 0–25 mM in the presence or absence of TNF-α for 24 h, and cell viability was examined using the MTT assay (Figure 2). The results suggest that 25 mM glucose is optimal for cellular growth, given the trend toward a progressive increase in viability as the mM glucose concentration increases, although these values did not attain between-group significance (Figure 2A). Exposure to TNF-α (10 ng/mL) resulted in the loss of metabolic activity. Metabolic failure (cessation) was reversed progressively in the presence of glucose and normalized with 25 mM glucose (*p* < 0.05) (Figure 2B).

### 3.2. High Glucose Reduced TNF-α-Induced IL-6 and IL-1β-Production in SH-SY5Y Cells

**Figure 3:**
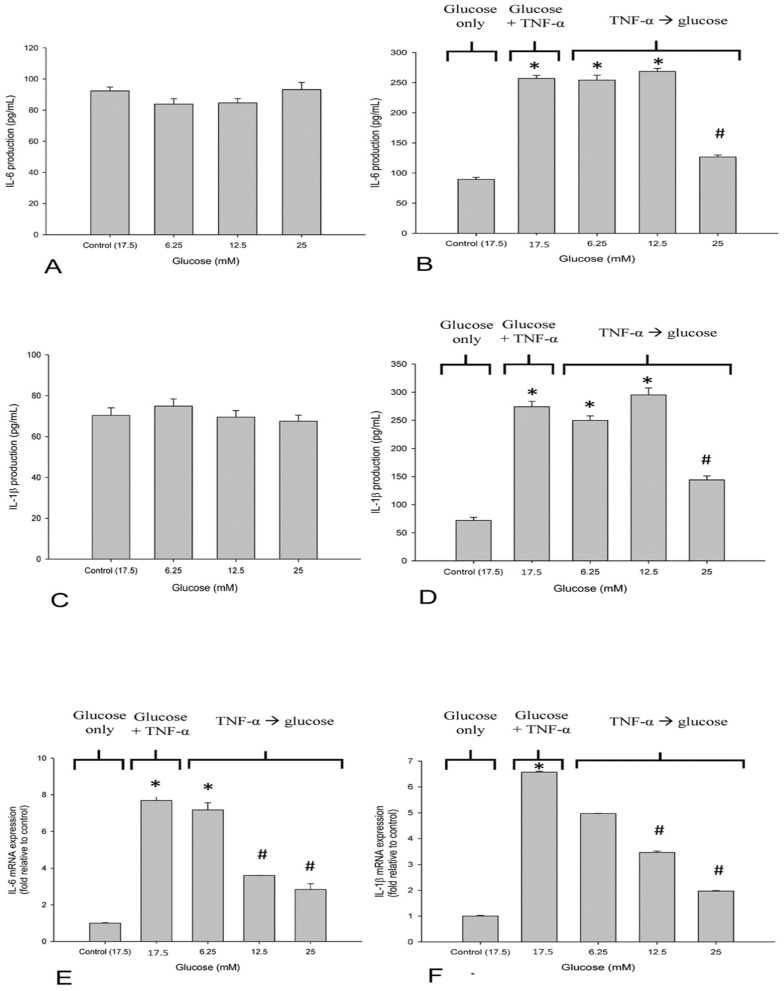
Effect of glucose on proinflammatory response in SH-SY5Y cells. Cells were treated with or without 10 ng/mL of TNF-α and cultured with different concentrations of glucose for 24 h. (**A**,**B**) IL-6 production was determined by ELISA. (**C**,**D**) IL-1β production was determined by ELISA. (**E**,**F**) mRNA expression of IL-6 and IL-1β upon TNF-α treatment was examined using qPCR. Data are expressed as mean ± SD. * *p* < 0.05 compared with 17.5 mM glucose control; ^#^ *p* < 0.05 compared with TNF-α + 17.5 mM glucose control.

We evaluated the effects of glucose on IL-6 production in neuronal SH-SY5Y cells in response to TNF-α exposure. Low baseline expression of IL-6 was detected in the supernatant of untreated neuronal SH-SY5Y cells. Treatment with glucose at different concentrations had slight effects on the baseline IL-6 levels in neuronal SH-SY5Y cells without a between-group significance (Figure 3A). Exposure to TNF-α also led to a 2.5-fold increase in IL-6 production in the SH-SY5Y cells. Elevated IL-6 production induced by TNF-α was reduced in response to 25 mM glucose treatment in SH-SY5Y cells (*p* < 0.05). In contrast, no significant changes in IL-6 production were observed at lower glucose concentrations (Figure 3B). We also investigated the influence of glucose on the production of TNF-α, which upregulates IL-1β, in neuronal SH-SY5Y cells. Increases in IL-1β expression were inhibited by 25 mM glucose treatment (*p* < 0.05) (Figure 3C,D). The upregulated expression of IL-6 and IL-1β was confirmed at the transcriptional level (*p* < 0.05) (Figure 3E,F).

### 3.3. High Glucose Reduced the Elevated mRNA Expressions of NF-κB and COX-2 in TNF-α-Treated SH-SY5Y Cells

**Figure 4:**
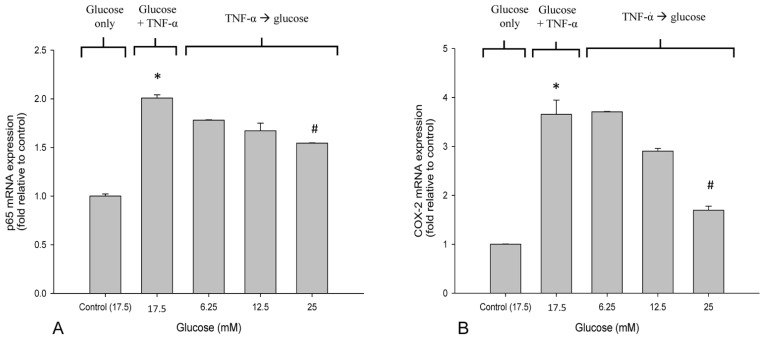
The effects of glucose on NF-κB p65 and COX-2 mRNA expression. SH-SY5Y cells were treated with TNF-α (10 ng/mL), followed by treatment with different concentrations of glucose for 24 h. (**A**) The mRNA expression of NF-κB p65 was measured with qPCR. (**B**) The mRNA expression of COX-2 was measured with qPCR. Data are expressed as mean ± SD. * *p* < 0.05 compared with 17.5 mM glucose control; ^#^ *p* < 0.05 compared with TNF-α + 17.5 mM glucose control.

The mRNA level of NF-κB p65 was upregulated in SH-SY5Y cells in response to TNF-α treatment (Figure 4A). The expression of COX-2 was significantly increased in SH-SY5Y cells by TNF-α treatment (Figure 4B). The results show that 25 mM glucose suppressed the elevated expression of NF-κB and COX-2 (*p* < 0.05), whereas no significant difference was found at lower glucose levels (Figure 4A,B).

## 4. Discussion

In the present study, we found that glucose restored TNF-α-inhibited cell growth and suppressed TNF-α-induced production of IL-6 and IL-1β in SH-SY5Y cells. Our study is the first to suggest that high glucose concentrations reduce inflammation-driven neurogenic deterioration. 

Except for potential sensorineural mechanisms, adenosine monophosphate protein kinase (AMPK) is a key enzyme that regulates cell metabolism in the body [41]. Decreased AMPK activity is related to many types of pain, including neuropathic pain. In contrast, activation of AMPK activity may reduce inflammatory reactions and pain levels [42]. When cells are in a hypoglycemic environment, AMPK activity may decrease, resulting in pain. Increasing glucose concentration in a timely manner may alleviate pain [42]. Other researchers have proposed that glucose indirectly inhibits the capsaicin-sensitive receptor (transient receptor potential vanilloid receptor-1, TRPV-1) and blocks the secretion of substance P and calcitonin gene-related peptide (CGRP), which are pro-nociceptive substances involved in neurogenic inflammation [43,44,45,46]. Interestingly, mannitol (a glucose analog) cream, when applied for lip pain caused by capsaicin, reduces pain significantly faster than vehicle cream, implying the indirect inhibition of the TRPV-1 receptor [44]. A direct effect has not been postulated because the TRPV-1 receptor has not been found to have a binding point for glucose or mannitol [47]. This study was designed and performed to contribute to a growing body of basic science literature focused on the effects of elevated glucose levels on nerve cells, specifically the effects on key cytokine production by those nerve cells in vitro. Our study is the first to support the hypothesis that glucose has an anti-neurogenic inflammatory effect, a potential mechanism of the therapeutic benefit of glucose when used for neuropathic pain, and should encourage further related research.

Accumulating evidence suggests that inflammation plays a role in the pathophysiology of neuropathic pain. During the inflammatory response to nerve injury, various inflammatory mediators such as cytokines and chemokines are released by damaged cells and immune cells in the microenvironment of the lesion, which in turn induces painful neuropathy [48]. TNF-α, a proinflammatory cytokine, has been suggested to be involved in the pathogenesis of neuroinflammation, and elevated TNF-α levels have been found locally and systemically in patients with neuropathic pain [49,50,51]. TNF-α signals through ligand binding to two receptors, TNFR1 and TNFR2. Binding of TNF-α to TNFR1 activates apoptosis via intracellular death domain proteins [52]. TNFR1 has been reported to be constitutively expressed in SH-SY5Y, whereas the level of TNFR2 was significantly low [53]. Our finding that TNF-α inhibited cellular metabolic state is consistent with the results of previous studies [53,54,55,56]. Previous studies have reported that a number of cell types have a limited capacity to regulate their glucose intake [57], and high glucose concentrations induce the production of pro-inflammatory mediators [58,59,60,61,62], including TNF-α and NF-κB transcription factors [57,60]. These effects are expected to worsen and not improve the metabolic status of cells already compromised by exposure to TNF-α. In contrast with other cell types studied such as macrophages, monocytes, or glial cells within or around nerve cells, there is no evidence that primary nerve cells produce TNF-α in response to elevated glucose levels [63,64]. In addition, it is notable that the standard glucose levels to maintain nerve cells (including SH-SY5Y cells) in culture are 17.5 mM or more, with no ill effects, and are tolerant of much higher glucose elevation than other cells [30,31,32,33,34]. Moreover, adult nerve cells may not become apoptotic despite substantial glucose elevation up to 60 mM [65]. In the present study, we found that a high glucose concentration (25 mM) was well tolerated in SH-SY5Y cells, showing no significant changes in cell metabolic function, which is consistent with previous studies [33,34].

TNF-α binding to TNFR1 upregulates the expression of several proinflammatory agents and induces NF-κB activation [66]. Exposure to exogenous TNF-α significantly upregulated the expression of IL-6 and IL-1β in SH-SY5Y cells. Our data showed that high glucose (25 mM) reduced the upregulated expression of IL-6 and IL-1β. It has been documented that TNF-α induces IL-6 production through phosphorylation of NF-κB in neuronal cells [67]. NF-κB, a transcriptional regulator, is involved in inflammation, cell proliferation, and cellular metabolic dysfunction/apoptosis. We could not confirm cellular apoptosis by MTT assay alone. Immunostaining for caspase-3, an essential component of neuronal cell death, is required to confirm apoptosis [68]. TNF-α binding results in the phosphorylation of inhibitory kappa B (IκB), dissociation of NF-κB from IκB, followed by nuclear translocation and subsequent transcription of several proinflammatory cytokines, including IL-6, IL-1β, and TNF-α [69,70]. We cannot confidently affirm that the reduction in cytokine expression in our study in the presence of glucose was based on the downregulation of NF-κB activity. Although a reduction in NF-κB production is indicated by a drop in mRNA levels for NF-κB, qPCR analysis for the Silent inhibitor of the Death Domain (SODD) or Western blotting to identify the phosphorylation status of NF-κB subunits would be important additional tests to confirm that NF-κB activity was directly reduced by glucose [71,72]. Nevertheless, NF-κB is a key regulator of COX-2 [73], and our findings of reduced cytokine levels and expression of COX-2 upon exposure to elevated glucose levels are both consistent with a potential reduction in NF-κB activity. Specifically, our results suggest that TNF-α-induced proinflammatory cytokine production is likely dependent on the NF-κB/IκB pathway, and that TNF-α-induced NF-κB activation likely mediates COX-2 expression in this neuronal cell type. The reduction in proinflammatory cytokines and inflammatory mediators by exposure to high glucose suggests a potential correction for cellular impairment (inflammation). Elevated levels of TNF-α have been documented to increase energy expenditure, promote energy updates, and potentially result in glucose deprivation [74,75,76,77,78]. We postulate that pretreatment with TNF-α elevation may initiate a glucose deprivation state, and that high glucose (25 mM) restores impaired glucose metabolism. However, we did not evaluate the mechanism by which high glucose (25 mM) restores SH-SY5Y cellular metabolic function in this study. Further studies focusing on energy regulators such as AMPK and the relationship between glucose and neurogenic inflammation may add to our understanding of high glucose-related protection of neuronal cell viability.

Although 5% glucose (equal to 5000 mg/dL) is approximately 50-fold higher than the physiological concentration in the plasma and tissue fluid, 5% glucose has a similar osmolality (277 mmol/L) as that of normal saline (308 mmol/L). Upon injection, it is less painful than sterile water [79]. Moreover, human and animal studies have shown that 5% glucose is not harmful to nerves [79,80,81], and no related adverse effects after hydrodissection with 5% glucose water have been reported [19,82]. In addition, 5% glucose is unlike hyperosmolar glucose (>10%), which characteristically initiates an inflammatory cascade and is used in prolotherapy to treat musculoskeletal pain [83]. No data are currently available to indicate how fast the concentration of 5% glucose drops in various body areas. Moreover, no local inflammation has been demonstrated after injection of up to 10% glucose, even in a space that might be expected to have a lower turnover, such as the carpal tunnel, suggesting that glucose levels may decline rapidly through several mechanisms [84]. During the preparation of our first randomized trial of 5% glucose versus saline injection in CTS, we observed that the fluid injected around the median nerve in the carpal tunnel was completely absorbed within 1 h, as detected by ultrasonography (Figure 4 in original text) [10]. Thus, we estimated that a 10-fold drop in glucose levels from 5% to 0.5% (27.8 mM) would occur within hours. Hence, we chose 25 mM glucose as the high glucose group in our study. Degradation studies based on the placement of dialysis needles are needed to confirm our choice of high glucose levels to optimally simulate clinical effects, and further research efforts to find the most suitable and effective glucose concentration for reducing proinflammatory cytokines and metabolic dysfunction in neural cells are important.

Our study has several limitations. First, although SH-SY5Y neuroblastoma cells are widely used in in vitro studies of many neurological disorders, they retain the characteristics of cancer cells. Cancer cells have an enhanced uptake of glucose and a more powerful response to glucose provision than non-cancer cells [85]. This limitation is mediated by enhanced uptake of glucose by neuronal cells [35]. SH-SY5Y, as with other cell cancer lines, may not enter true apoptosis (initiation of a programmed cell death cycle) in response to TNF-α, since cancer cell lines are “immortalized” (i.e., do not have a death program) [86]. From this perspective, TNF-α-induced loss of MTT activity indicated, at a minimum, marked metabolic cessation in SH-SY5Y cells [87]. Regardless of whether SH-SY5Y cells were classically apoptotic, glucose was able to restore the metabolic function of the cells and favorably impact their production of inflammatory cytokines [88]. It would be optimal to repeat these studies with other neural cell types in the future. Second, the extracellular milieu in peripheral nerves was not replicated in an in vitro neuroblastoma cell culture model. This study reports an association between glucose elevation and protective effects on a neural cell line challenged by TNF-α exposure, reflected by the restoration of metabolism and reduction of inflammatory mediators, although we cannot confirm that this was due to reduced activation of NF-κB. These results can be suggestive only and cannot be considered as an accurate understanding of the effect of dextrose injection on peripheral entrapment neuropathy. In addition to in vitro approaches to understand the apparent ameliorative effect of glucose elevation in compression neuropathy, an in vivo approach, in which microdialysis needles are utilized to measure changes in cytokines, COX-2, SODD, NFκB phosphorylation status, etc., may be particularly useful in advancing knowledge of the effects of glucose elevation [89].

## 5. Conclusions

The present study provides evidence that TNF-α-induced neural cell metabolic dysfunction and inflammation are likely mediated by the activation of the NF-κB pathway. High glucose levels can mitigate TNF-α-induced NF-κB activation, the upregulation of proinflammatory cytokines, and metabolic dysfunction in neural cells.

## Figures and Tables

**Table 1:** Primers used in the PCR.

IL-6	5′-TAGCCCTGAGAAAGGAGACATG-3′	5′-AGGCAAGTCTCCTCATTGAATC-3′
NF-κB	5′- CAAGAAGTCCACAAACAC-3′	5′- ACCGATATGTCCTCTTTC -3′
COX-2	5′-AACATCGTCAATAGCATTC-3′	5′-AACATCGTCAATAGCATTC-3′
IL-1β	5′-AGAAGCTTCCACCAATACTC-3′	5′-AGCACCTAGTTGTAAGGAAG-3′
β-actin	5′-TGACGTGGACATCCGCAAAG-3′	5′-CTGGAAGGTGGACAGCGAGG-3′

## Data Availability

The data presented in this study are available on request from the corresponding author.
